# Ablative radiofrequency microplasma for lateral dermatochalasis-indications and treatment recommendations

**DOI:** 10.1007/s10103-024-04061-8

**Published:** 2024-12-18

**Authors:** Arie Y. Nemet, Efrat Solomon-Cohen, Anna Aronovich, Malachy Nemet, Daniel Hilewitz, Achia Nemet, Kaplan Baruch, Lehavit Akerman

**Affiliations:** 1 Department of Ophthalmology, Meir Medical Center, 59 Tshernichovsky St., 4428164 Kfar Saba, Israel; 2 Sackler School of Medicine, Tel Aviv University, Tel Aviv, Israel; 3 Division of Dermatology, Rabin Medical Center, Petach-Tikva, Israel; 4 Clalit Health Services, Dan-PT District, Israel; 5 Assuta Ashdod Medical Center, Ashdod, Israel; 6 Adelson School of Medicine, Ariel University, Ariel, Israel

**Keywords:** Ablative Radiofrequency, Microplasma, Dermatochalasis, Blepharoplasty

## Abstract

To determine in which cases ablative radiofrequency microplasma is preferred for the treatment of lateral dermatochalasis over a surgical approach as well as discussing each method's benefits and limitations. Twenty-one patients underwent 3 interventions of plasma exeresis. Photographic and RCM images were acquired at baseline and 4 weeks after final plasma exeresis. The eyes were categorized into 3 groups based on the dermatochalasis severity (1- mild, 2- moderate, 3- severe). Additionally, a further division was conducted to assess the degree of enhancement observed after the treatment (1- slight improvement, 2- moderate improvement, 3- significant improvement). The classifications and assessments were performed by was graded by two trained dermatologists as blinded observers. A total of 21 eyes with a mean age of 54 years (range45-67 years) and 100% females were included in this study. The severity of dermatochalasis directly affects the clinical improvement (*P*=0.039) and the higher the severity, the more the improvement (R = -0.62). Noninvasive ablative microplasma may offer safe and effective therapy for upper eyelid dermatochalasis and can even be performed in patients at surgical risk. However, it may be suitable for grades 0 and 1 of DC. For more advanced grades a surgical solution achieves better results for the treatment of dermatochalasis of the upper eyelid.

## Introduction

Dermatochalasis (DC) is defined as a redundancy and laxity of the eyelid skin and muscle. Age- and ultraviolet-induced degeneration of the skin causes a loss of elasticity in the connective tissue of the eyelids, resulting in DC [1]. Signs of DC may begin around age 40 and continue to progress with age [2], but is occasionally seen in young adults [3].

The frontal region, the eyebrows, and the upper eyelids exhibit age related changes in their structure [4]. Eyelid DC is considered with its lateral extension in the temporal region as an aesthetic and functional unit [4–6], and changes of DC must be considered as part of the whole complex of the upper face.

DC can overlap the whole upper eyelid and, by gravity, leads to mechanical ptosis of the upper eyelid resulting in upper and lateral visual field disturbances as well as other clinical manifestations [6]. Most commonly, DC is a cosmetic concern as patients note a fullness or heaviness of the upper eyelids which may lead to an older appearance [7]. Temporal or lateral dermatochalasis has been described as lateral hooding, lateral heaviness, and lateral drooping [5, 8–10].

Until recently, treatments for all types of DC and the aging of the periorbital area were surgical blepharoplasty, lasers or filler injections [11]. Surgical blepharoplasty is still the main requested procedure for eyelid dermatochalasis, especially for severe cases, and has been associated with excellent results in expert centers [12].

In recent years there has been a gradual tendency and demand for non-surgical solutions using mainly energy-based devices. Several previous studies demonstrated that a plasma radiofrequency ablation technology for upper blepharoplasty could be recommended in mild and moderate DC without lipotosis [13–17].

However, researching the current literature, no conclusive guidelines or algorithm has been published to date. In the current study we retrospectively analyzed cases of DC by both an oculoplastic surgeon and aesthetic dermatologists. The purpose of this study was to determine in which cases the surgical approach is recommended over plasma exeresis as well as to discuss each method's benefits and limitations.

## Materials and methods

### Patients

Patient inclusion criteria included: 1) photo skin Type I–IV 2) the presence of moderate to severe laxity of the eye fold, according to the facial laxity rating scale. Exclusion criteria were: 1) patients with eyelid ptosis due to muscular or neurological impairments 2) possible impaired wound healing, or 3) patients with skin integrity compromise.

Twenty-one healthy subjects, all females, aged 45 to 67 (mean age of 54), with mild to severe upper eyelid dermatochalasis (DC) were included in the study.

### Treatment

Following gentle cleansing of the treated area, a topical anesthetic cream (Lidocaine 8%) was applied for 60 min. The area was then revealed and disinfected with benzalchonium.

Nonsurgical blepharoplasty was carried out with the FDA approved high power Pixel RF Colibri tip (Accent Prime Alma Lasers. GmbH, Germany) which induces controlled ablative micro-plasma sparks and a deeper thermal modified (coagulation) area. Ablation micro-injuries of the upper eyelid were focused over the lid crease and below the eyebrow, producing a crescent-shaped ablative area.

Treatment parameters included: power levels of 5W at high ablation phase (phase 4) and thermal phase (phase 1), and a 4–6 crescent-shaped passes over the treatment area. Treatments consisted of 1 to 3 sessions at 2 months intervals and follow-up visits at 2 months following the final treatment.

Written informed consent and signed picture and photo release agreement were obtained from each patient. Patients were treated according to regional laws and good clinical practice following a standard protocol.

Post-treatment care included: washing the treatment area with neutral soaps only, disinfect the area as well as application of topical fucidic acid 3 times daily for 7 days post treatment.

Digital photographs were taken at baseline and at each follow-up visit. Safety was evaluated by assessment of adverse events after each treatment. Patients were also instructed to monitor for adverse events or post treatment complications.

### Clinical evaluation

DC severity was graded by two trained dermatologists as blinded evaluators.

**Fig. 1:**
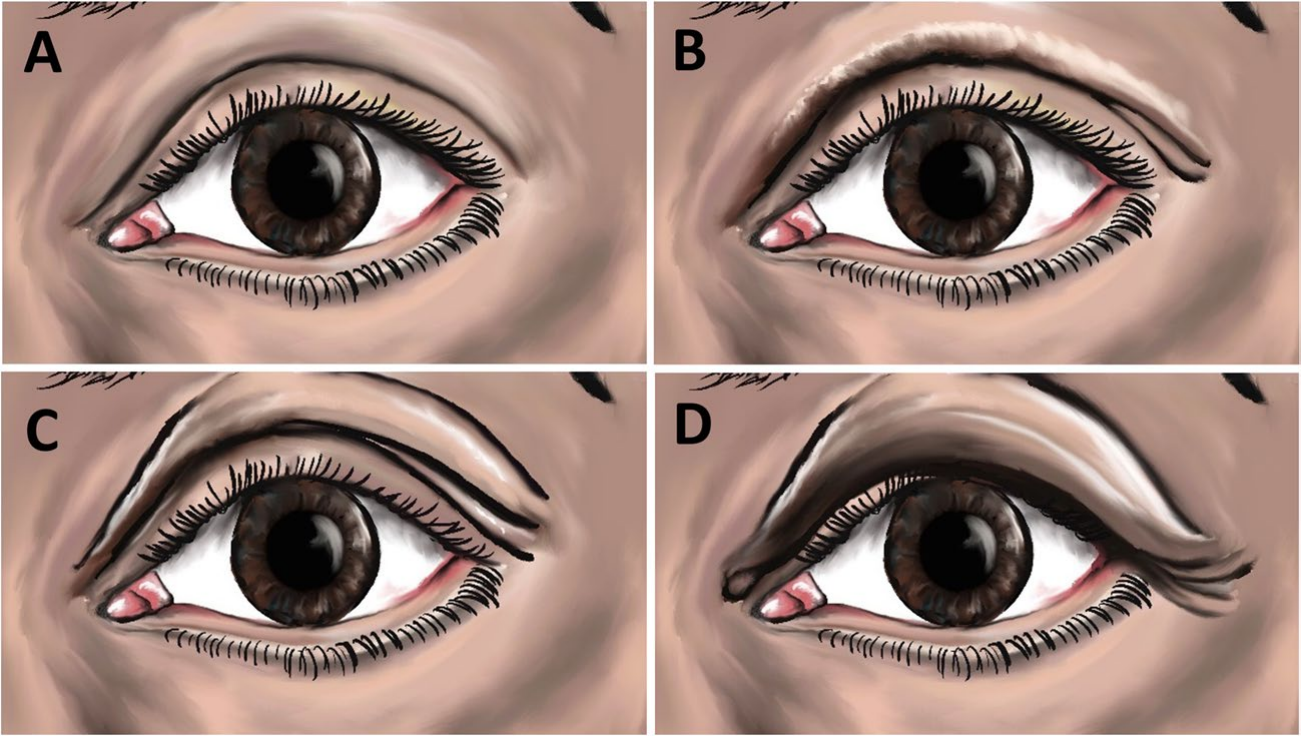
Dermatocholasis severity grading scheme. DC severity scored by a 4 point scale: A, grade 0 = no excess skin, B, grade 1 = mild overhang skin, C, grade 2 = moderate overhang over lid and crease, and D grade 3 = severe overhang

In order to evaluate the dermatochalasis, we used the lateral dermatochalasis scales (LDC) classification (Fig. 1), which includes four degrees, as described recently by Silva et al. [4].

- Grade 0—absence of DC, in the lateral region of the orbit.
- Grade 1—lower edge of DC is located above the intersection of the lacrimal caruncle with the edge of the upper eyelid.
- Grade 2—between the intersection of the lacrimal caruncle with the edge of the upper eyelid and the lower edge of the iris at the pupillary midpoint, even when the lower DC is at the same level of the intersection of the lacrimal caruncle with the edge of the upper eyelid.
- Grade 3— lower DC below the lower edge of the iris, even when it is at the same level of it.

Results were evaluated blindly by two physicians 30 days post-procedure.

## Results

A total of 21 eyes with a mean age of 54 years (range, 45–67 years) and 100% females were included in this study.

The eyes were categorized into 3 groups based on the dermatochalasis severity (1- mild, 2- moderate, 3- severe). Additionally, a further division was conducted to assess the degree of enhancement observed after the treatment (1- low improvement, 2- intermediate improvement, 3- significant improvement). The classifications and assessments were performed by an oculoplastic specialist. Table 1, summarizes the distribution of cases according to severity and according to clinical improvement.

**Table 1:** The distribution of clinical improvement of ablative radiofrequency according to the severity of dermatochalasis

Dermatochalasis grade	Grade 1 (mild)	Grade 2 (moderate)	Grade 3 (severe)
Improvement grading			
1 = low improvement	10% (1)	25% (1)	42.9% (3)
2 = intermediate improvement	0% (0)	25% (1)	42.9% (3)
3 = significant improvement	90% (9)	50% (2)	14.3% (1)
Total eyes (21)	(10)	(4)	(7)

**Fig. 2:**
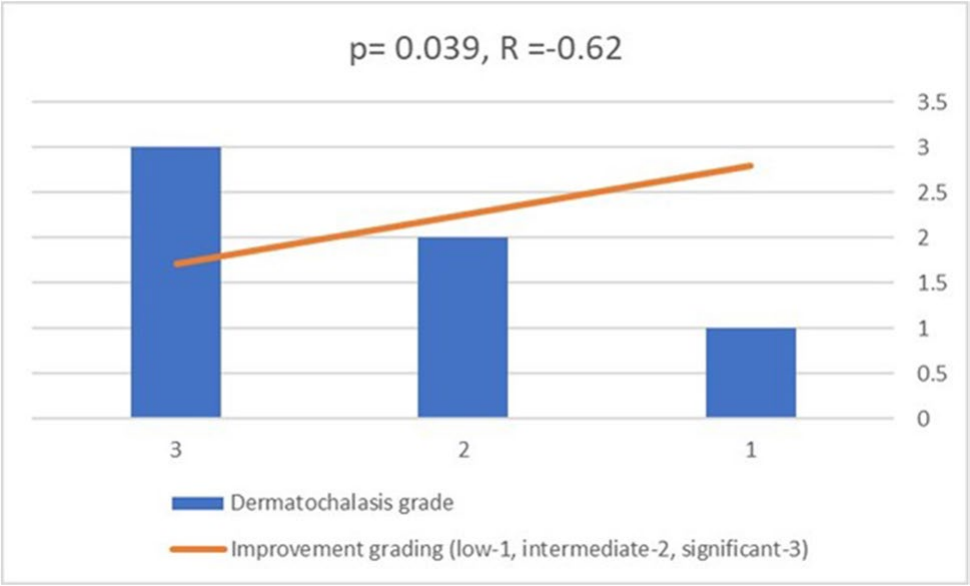
Correlation between the severity of dermatochalasis and clinical improvement. There is a significant correlation between the severity of dermatochalasis and clinical improvement, as indicated by a Pearson correlation coefficient of [R = -0.62, p = 0.039], suggesting that as the severity of dermatochalasis increases, the clinical improvement also increases

It can be seen that the severity of dermatochalasis directly affects the clinical improvement (*P*=0.039) and the higher the severity, the more the improvement (R = -0.62). (Fig. 2).

## Discussion

Dermatochalasis (DC) is associated with eyelid skin elastic tissue loss and with impaired local lymphatic drainage [18]. Elastic fibers play an important structural and functional role in lymphatic drainage with clinical or subclinical inflammation leading to loss of elastic tissue and low- lymphatic output may well explain the phenomenon of “baggy” eyelids [19].

A significant familial, racial and ethnic anatomical differences exist in eyelid anatomy. "Double” and “single” eyelids, epicanthi and palpebral fissure slant, fuller or deeper sulcus, different composition of fatty tissue and variations of upper lid fold, making the distinction between different eyelids very important when taking surgical considerations [20].

Several techniques for treating DC are used including invasive and non – invasive treatments such as blepharoplasty and laser therapy [21]. Technique selection mainly depends on: the degree of DC surgical risk, preferences of the patient and experience of the operator [4].

DC of the temporal region is well classified by the lateral DC classification [4]. This classification uses well-defined references of anatomical points, and may be assessed in frontal digital photographs, using horizontal lines as parameters to certify the correct anatomical points.

A pivotal feature of the blepharoplasty is its extension to the lateral region of the eyelid, until reaching the anatomical point three of the LDC, and the possibility of its association with other treatments, such as the suspension of the eyebrows and facial lifting [4].

Plasma skin regeneration is a novel type of skin rejuvenation technology [22] developed in recent years. The technology can present itself in four different phases: solid, liquid, gas, and plasma. By adding heat or energy to a gas, this is transformed into plasma: the atoms that make up the gas begin to lose their electrons and become positively charged ions. The lost electrons are then able to float freely and ionized. When this process involves most of the gas it is referred to as 'plasma' [23]. Devices using plasma technology deliver thermal energy directly to the tissues at the time of contact.

Hence, the two methods are different- surgery includes excision of the skin, some of the orbicularis muscle, and the prolapsed fat tissue. Plasma emesis is a non-invasive skin tightening procedure. Skin fibers contract and tighten, creating a lifting, remodeling, and rejuvenating effect. Plasma induces a denaturation of collagen and other proteins in the skin and follows a cascade of neo-collagenization, disruption of dermal solar elastosis, fibroblast activation and migration from the deeper dermis and cytokine release [17].

Previous studies have claimed that plasma radiofrequency ablation technology upper blepharoplasty could be recommended in mild and moderate DC without lipotosis [13, 14].

In this retrospective study we have found that in lateral DC plasma skin regeneration demonstrates good results in DC grade 0 to 1. Its' advantages include: no scaring, less tendency of hemorrhage, no incisions and stitches, shorter downtime, local anesthesia alone, no need for a whole surgical setting with lower patient costs, with yet desirable effects for the patient. However, from our experience in the following conditions non-satisfying results were demonstrated: lateral brow hooding, grade of excess skin is 2 or more, fat prolapse under the skin. In these conditions a surgical approach is likely to result in a better outcome, therefore patients who prefer a non-surgical approach should be set with realistic expectations, accordingly.

Naturally, these questions are addressed differently also according to the practitioner performing the treatment. A dermatologist would be prone to non-surgical solution, and the plastic or oculoplastic surgeon would likely tend to favor a surgical solution.

In our study we included two highly experienced esthetic physicians—one is dermatologist (LA), and the other in an oculoplastic surgeon (IAN).

### Grading of dermatochalasis

For the purposes of the paper, we used the lateral dermatochalasis scales (LDC) classification of Silva etal [4] as it measures the main point of activation performed either surgically or by the plasma. Thus far, several scales have been developed to grade dermatochalasis of the upper eyelid [24–26].

The following are points to be taken into consideration for the decision of preferable method- surgical or non-surgical treatment.

### The brow position

Brow ptosis is an important part of the preoperative assessment of blepharoplasty patients, especially when dealing with cases of lateral DC. When the brow descends, it compresses the soft tissues of the eyelid and weighs them down, often causing excess skin to prolapse over the lid margin and contact the eyelashes, a condition known as pseudoptosis.

When ptotic eyebrows accompany dermatochalasis, however, they often accentuate the upper eyelid abnormality and should be taken into consideration during surgery [27].

Patients with significant ptosis, heavy brows, medial greater than lateral ptosis, or post-facial palsy might not be good candidates for this procedure [28].

In such cases plasma treatment may be better, as it does not pull down the tissue. A subset of patients will manifest latent brow ptosis after eyelid surgery. It has been shown that ptosis surgery, whether done via Muller’s muscle conjunctival resection or by external levator advancement, leads to a decrease in brow height [29]. In cases of blepharoplasty alone, the change in brow height in studies are mixed [30]. Ptosis of the tail of the brow is the most frequently encountered brow deficit [31].

Eyebrows and eyelids vary among races, ages and genders. It is clear that each case is different and that adjusting the position of the eyelids and brows is complex, with interplay between muscles and connective tissue structures with neuronal pathways and interactions.

### Safety

Safety of surgical solution are remarkably high, and complications are uncommon [12], however, there are possible complications mainly dry eye syndrome, corneal abrasion, cellulitis, asymmetry and scarring [32].

The safety of plasma exeresis in an animal model was previously demonstrated. Plasma has been shown to limit the damage within the connective parenchyma, enabling faster healing, both in the immediate and postoperative reparative processes as compared with electrosurgical/radio scalpel therapy [17].

### Down time

For plasma exeresis one to three treatment sessions are needed at 2 months interval to achieve the optimal result. Patients should plan on 2–4 days down time and it takes about 12 weeks to see final results. In surgical blepharoplasty the healing process is significantly longer with bruising and edema around the eyelids. One can expect to spend approximately 2–3 weeks of resting and recovering.

### Cost and Cover by insurance companies

Dermatochalasis is a unique condition as it is an aesthetic issue in its early stages and may develop later on to a medical indication for treatment. Eyelid surgery should improve abnormal function, reconstructs deformities, or enhances appearance and may be either reconstructive or cosmetic (aesthetic).

Blepharoplasty of the upper eyelids is considered reconstructive when it provides functional vision and/or visual field benefits or improves the functioning of a malformed or degenerated body member, but cosmetic when done to enhance aesthetic appearance. Medicare does not cover cosmetic surgery or expenses incurred in connection with such surgery [33]. In severe cases of DC, usually grade 3 in the measured stage we have used, Medicare would cover the surgery cost. In contrast, Plasma is never covered by the health insurance.

## Conclusions

The plasma exeresis was an efficient treatment for DC graded 0 to 1. A significant improvement in the appearance of the eyelids is appreciated: However, for more advanced grades microplasma due to the noninvasive nature, non-surgical therapy of the eyelid skin is often favored and can even be performed to patients at surgical risk. However, we found that from the aesthetic point it may be suitable just for the grades 0 and 1 of DC- the DC is considerably reduced, the periocular wrinkles are smoothed, and the eyes take on a more youthful appearance. For more advanced grades of 2 or 3, in cases of lateral brow hooding or in eyelid fat prolapse the results of plasma exeresis were unsatisfactory. In such cases a surgical solution achieves better results for the treatment of DC of the upper eyelid. Proposed algorithm is summarized in Table 2.

**Table 2:** Principles for Lateral Dermatochalasis Management Algorithm

DC graded 0 to 1	The plasma exeresis is an efficient treatment
DC graded 2 to 3	Blepharoplasty is the preferred choice
For lateral brow hooding grade ≥ 2	Blepharoplasy with brow lift
